# Efficacy of Natural Formulation Containing Activated Charcoal, Calcium Sennosides, Peppermint Oil, Fennel Oil, Rhubarb Extract, and Purified Sulfur (Nucarb®) in Relieving Constipation

**DOI:** 10.7759/cureus.18419

**Published:** 2021-10-01

**Authors:** Rashid Ali, Muhammad Irfan, Umair Akram, Maroof Vaince, Kamran Hassan, Adnan Maqsood, Ambreen Aslam, Nabeel Amaan, Adeel Qamar, Sidra Memon

**Affiliations:** 1 Internal Medicine, Mayo Hospital, Lahore, PAK; 2 Internal Medicine, Allied Hospital, Faisalabad, PAK; 3 Internal Medicine, Lady Reading Hospital Medical Teaching Institute, Peshawar, PAK; 4 Internal Medicine, Ganga Ram Hospital, Lahore, PAK; 5 Family Medicine, Gujrat Hospital, Gujrat, PAK; 6 Internal Medicine, Sahara Hospital, Narowal, PAK; 7 Internal Medicine, Jinnah Sindh Medical University, Karachi, PAK

**Keywords:** natural medicine, nucarb, constipation, tenesmus, laxatives

## Abstract

Introduction

Long-term use of laxatives may have side effects such as bloating, allergic reaction, abdominal pain, metabolic disturbances, and hepatotoxicity. In this study, we have compared the efficacy of herbal medicine Nucarb, a combination of activated charcoal, calcium sennosides, peppermint oil, fennel oil, rhubarb extract, and purified sulfur, in relieving constipation.

Methods

This longitudinal study was conducted in multiple cities of Pakistan from April 2021 to June 2021. A total of 1000 patients, of either gender between age group 18 and 75 years, with complete spontaneous bowel movement of less than or equal to two times per week, were enrolled in the study. Participants were prescribed two tablets of Nucarb once daily (OD) at bedtime for the first seven days, followed by one tablet of Nucarb OD at bedtime for the following seven days. They were asked to return for follow-up after 14 days.

Results

There was a statistically significant improvement in all six components of constipation. After 14 days, the severity of constipation reduced by 80.70%, the sensation of straining was reduced by 72.69%, and the feeling of incomplete evacuation was reduced by 71.87%. There was no adverse event reported.

Conclusion

Nucarb is efficacious in reducing the severity of constipation, sensation of straining, bloating and abdominal pain, feeling of incomplete evacuation, and difficulty in passing gas. Since it is a herbal product, it can be safely used in all populations.

## Introduction

Constipation is defined as the disorder of motility of the gastrointestinal tract, which includes symptoms such as infrequent stools, difficult stool passage with pain and stiffness. In cases of acute constipation, intestine may get close, which may even require surgery [1]. Constipation is a global issue, with prevalence reported as high as 80% and resulting in much costs for the community [2]. There are various factors responsible for constipation. These factors include genetic predisposition, socioeconomic status, low fiber consumption, lack of adequate fluid intake, lack of mobility, disturbance in the hormone balance, side effects of medications, and anatomy of the body [1].

Primary management of constipation should consist of lifestyle modifications, reassurance of their concept of a healthy or “regular” bowel movement, and biofeedback [3]. In cases where symptoms do not improve with non-pharmacologic management, laxatives should be the next line of treatment for the management of constipation [3]. Various adverse effects are reported with long-term use of laxatives. These adverse events can be mild such as bloating, allergic reaction, and abdominal pain or severe such as metabolic disturbances and hepatotoxicity [4]. Furthermore, the multifactorial causes of constipation restrict the clinical efficacy of current conventional Western treatments as these drugs act through a single pathway [5]. To help overcome these shortcomings and deliver a complete holistic approach, herbal medication with the ability to target multiple organ sites may be used [6].

The purpose of our study is to compare the efficacy of herbal medicine Nucarb (a combination of activated charcoal, calcium sennosides, peppermint oil, fennel oil, rhubarb extract, and purified sulphur) in relieving constipation.

## Materials and methods

This longitudinal study was conducted in multiple cities of Pakistan from April 2021 to June 2021. A total of 1000 patients, of either gender between the age group 18 and 75 years, with complete spontaneous bowel movement of less than or equal to two times per week, were enrolled in the study. Patients with drug-induced constipation or constipation due to secondary causes were excluded from the study.

After informed consent, detailed history of the patients, including the number of stools in the last week and drug history, was taken, and they were asked to fill a self-structured questionnaire. The questionnaire had a visual analog scale (VAS) where participants were asked to rank the severity of constipation, feeling of incomplete evacuation, the sensation of straining, bloating, and abdominal pain, and difficulty in passing gas from zero to four (four being worse and zero being no symptoms). The questionnaire was explained to the participants to ensure that they completely understood each question. Participants were prescribed two tablets of Nucarb once daily (OD) at bedtime for the first seven days, followed by one tablet of Nucarb OD at bedtime for the next seven days. They were asked to return for follow-up after 14 days. In the follow-up, patients were asked to fill VAS scale again for all six elements. Any adverse event was also reported in the questionnaire. We lost 99 participants to follow-up. Only participants who completed the follow-up period of 14 days were included in the study.

Data were analyzed using Statistical Package for Social Sciences® software (version 23.0; SPSS; IBM Corp., Armonk, NY, USA). Numerical variables and data were presented as mean and standard deviations. Categorical values were tabulated as frequencies and percentages. The t-test was used to compare the participant’s responses on day 0 and day 14. A p-value of less than 0.05 means there is a significant difference between the two groups.

## Results

A total of 901 participants completed the study. Most participants were between 46 and 60 years (44.6%). Male participants (67.9%) were more common in our study (Table 1).

**Table 1:** Demographics of the participants

Characteristics	Frequency (%)
Age group	
18-30	56 (6.2%)
31-45	92 (10.2%)
46-60	402 (44.6%)
61-75	351 (38.9%)
Gender	
Male	612 (67.9%)
Female	289 (32.1%)

There was a statistically significant improvement in all six components of constipation. After 14 days, the severity of constipation reduced by 80.70%, the sensation of straining was reduced by 72.69%, and the feeling of incomplete evacuation was reduced by 71.87% (Table 2).

**Table 2:** Comparison of severity score of symptoms on day 0 and day 14

Symptoms	Severity score		Decrease in severity (%)	p-Value
	Day 0	Day 14		
Difficulty in passing gas	2.85 ± 0.89	0.55 ± 0.42	80.70	<0.0001
Sensation of abdominal pain	2.82 ± 0.72	0.77 ± 0.51	72.69	<0.0001
Sensation of bloating	2.56 ± 0.82	0.72 ± 0.61	71.87	<0.0001
Incomplete evacuation	2.35 ± 0.65	1.07 ± 0.62	54.46	<0.0001
Sensation of straining	2.31 ± 0.62	0.72 ± 0.51	68.83	<0.0001
Severity of constipation	2.47 ± 0.61	0.82 ± 0.53	68.80	<0.0001

## Discussion

In our study, most of the patients with constipation were included in the age group 46-60 (44.6%) and were male (67.9%). After intake of Nucarb OD for a span of 14 days, difficulty in passing gas was significantly reduced (80.7%), followed by reduced abdominal pain (72.69%) and bloating (71.87%). Moreover, considerable relief in the intensity of constipation (68.80%), incomplete evacuation (54.46%), and sensation of straining (68.83%) was also observed.

The possible explanation for the relief in constipation is due to the laxative property of the components included in Nucarb. Calcium sennoside, an anthraquinone drug, contains inactive glycosides. When glycosides are taken, they do not undergo any change in the small intestine. They are broken down by the bacterial glycosidases present in the colon to form active molecules. These molecules allow the entry of electrolytes into the colon and trigger myenteric plexuses to initiate peristalsis in the bowel [3]. After the oral intake of anthraquinones, the passage of stool is observed within 6-8 hours. Controlled trials have proved that senna can soften stools and stimulate bowel movements to induce frequent defecation [3]. Senna and magnesium oxide have been shown to significantly improve the frequency of bowel movements and quality-of-life score and seem to be effective in the treatment of constipation [7]. Rhubarb extract has also been shown to cause beneficial effects in constipation. It contains tannin that can impose antidiarrheal effects [8].

Other agents used for relieving constipation are magnesium and sulfate ions. Magnesium sulfate is a strong laxative that is used to distend the stomach and cause a large amount of liquid stool [9]. Sodium sulfate is also used for colonic irrigation for diagnosis and before surgery [10,11]. Peppermint oil has several mechanisms of action including smooth muscle relaxation via calcium channel blockade or direct enteric nervous system effects, visceral sensitivity modulation via transient receptor potential cation channels, and psychosocial distress modulation. It also has antimicrobial and anti-inflammatory activity [12]. The use of fennel oil is associated with a decrease in colic pain [13]. Various studies have proven the safety of herbal medicines for constipation [8,12,14,15]. No serious adverse event was reported in our study.

To the best of our knowledge, this is the first study that targets the efficacy of the combination of herbal medicine for constipation. Since the study was conducted in various cities and included both genders and participants of all age groups, data generated can be considered reliable and provide a basis for further investigation regarding the role of herbal medicine in constipation. Since the study has only single arm, care should be taken while comparing the result against other molecules or formulations.

## Conclusions

Our study indicates that herbal medicines, such as Nucarb used in this study, are an effective way of managing constipation. Herbal medicine can be used as first line for constipation or alternative in patients experiencing adverse events on pharmacological management. Further large-scale studies are needed to validate the findings of our result.
